# Tetra­kis[μ-2-(3-phenoxy­phen­yl)propionato-κ^2^ *O*:*O*′]bis­[(dimethyl­formamide-κ*O*)copper(II)]

**DOI:** 10.1107/S1600536808038786

**Published:** 2008-11-26

**Authors:** Mariela A. Agotegaray, Oscar V. Quinzani, Ricardo Faccio, Cecilia Goyenola, Álvaro W. Mombrú

**Affiliations:** a Departamentode Química, Universidad Nacional del Sur, Av. Alem 1253, B8000CPB, Bahía Blanca, Argentina; b Crystallography, Solid State and Materials Laboratory (Cryssmat-Lab), DETEMA, Facultad de Química, Universidad de la República, CC 1157, 11800 Montevideo, Uruguay; c Centro NanoMat, Polo Tecnológico de Pando, Facultad de Química, Universidad de la República, 91000, Canelones, Uruguay

## Abstract

The title compound, [Cu_2_(C_15_H_13_O_3_)_4_(C_3_H_7_NO)_2_], is formed by the chelate coordination of four racemic fenoprofenate (fenoprofenate is 2,3-phenoxyphenyl propionate) anions and two dimethyl­formamide mol­ecules to two copper(II) ions, building a paddle-wheel dinuclear mol­ecule. The distorted square-pyramidal coordination of each Cu^II^ atom is made up of four O atoms of the four fenoprofenate units and another O atom from a dimethyl­formamide mol­ecule. The two enanti­omeric forms of the fenoprofenate anions are present in the complex, in an optically inactive centrosymmetric arrangement.

## Related literature

For the properties of fenoprofen, see: Brogden *et al.* (1977 ▶); Nickander *et al.* (1977 ▶); Weder *et al.* (2002 ▶). For fenoprofen structures, see: Hamilton & Chen (1988*a* ▶,*b* ▶); Stephenson & Diseroad (2000 ▶); Weder *et al.* (2002 ▶); Zhu *et al.* (2001 ▶).
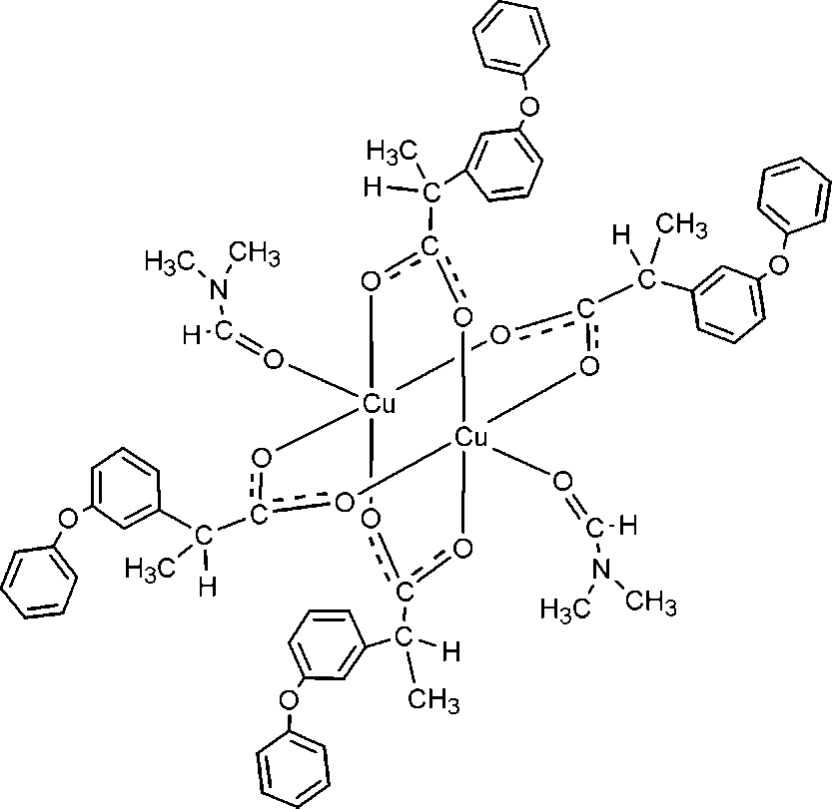


## Experimental

### 

#### Crystal data

[Cu_2_(C_15_H_13_O_3_)_4_(C_3_H_7_NO)_2_]
*M* *_r_* = 1238.29
Monoclinic, 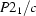

*a* = 11.142 (8) Å
*b* = 11.580 (8) Å
*c* = 23.891 (6) Å
β = 99.85 (6)°
*V* = 3037 (3) Å^3^
*Z* = 2
Mo *K*α radiation
μ = 0.77 mm^−1^
*T* = 293 (2) K
0.20 × 0.20 × 0.10 mm

#### Data collection

Rigaku AFC-7S diffractometer
Absorption correction: ψ scan (North *et al.*, 1968 ▶) *T*_min_ = 0.862, *T*_max_ = 0.927
8818 measured reflections
6969 independent reflections
4101 reflections with *I* > 2σ(*I*)
*R*_int_ = 0.091
3 standard reflections every 150 reflections intensity decay: none

#### Refinement

*R*[*F* ^2^ > 2σ(*F* ^2^)] = 0.064
*wR*(*F* ^2^) = 0.200
*S* = 1.02
6969 reflections
381 parameters
H-atom parameters constrained
Δρ_max_ = 0.51 e Å^−3^
Δρ_min_ = −0.54 e Å^−3^

### 

Data collection: *MSC/AFC Diffractometer Control Software* (Molecular Structure Corporation, 1993 ▶); cell refinement: *MSC/AFC Diffractometer Control Software*; data reduction: *MSC/AFC Diffractometer Control Software*; program(s) used to solve structure: *SHELXS97* (Sheldrick, 2008 ▶); program(s) used to refine structure: *SHELXL97* (Sheldrick, 2008 ▶); molecular graphics: *DIAMOND* (Brandenburg, 2006 ▶); software used to prepare material for publication: *PLATON* (Spek, 2003 ▶).

## Supplementary Material

Crystal structure: contains datablocks I, global. DOI: 10.1107/S1600536808038786/bg2218sup1.cif

Structure factors: contains datablocks I. DOI: 10.1107/S1600536808038786/bg2218Isup2.hkl

Additional supplementary materials:  crystallographic information; 3D view; checkCIF report

## Figures and Tables

**Fig. 1.**
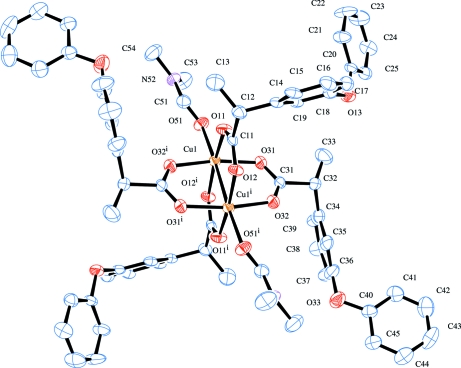
The structure of the Cu2(C15H13O3)4(C3H7ON)2complex with the labelling scheme. Displacement ellipsoids are drawn at the 30% probability level. Hydrogen atoms have been omitted for clarity.

**Fig. 2.**
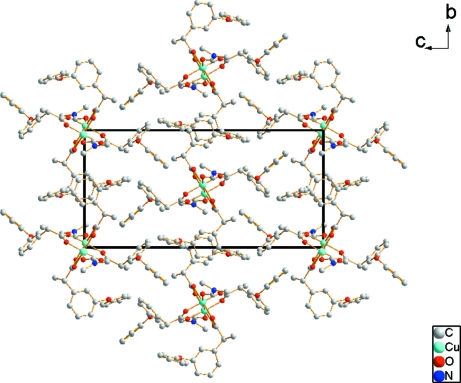
A view of the packing scheme, normal to the [100] direction.

## supplementary crystallographic information

### Comment

Fenoprofen[2-(3-phenoxyphenyl)propionic acid] is a non steroidal anti-inflammatory, antipyretic and analgesic drug (Nickander *et al.*, 1977; Brogden *et al.*, 1977). Its crystalline structure as a pure compound is not known. The crystal structures of the inclusion complexes of β-cyclodextrin with racemic fenoprofen and its enantiomerically pure forms have been reported: β-cyclodextrin(*RS*)-fenoprofen clathrate hydrate, β-cyclodextrin (*R*)-(-)-fenoprofen clathratehydrate, and β-cyclodextrin (S)-(+)-fenoprofen clathrate hydrate (Hamilton & Chen, 1988a,1988b).

Little is known about chemical structures of fenoprofen salts and complexes. Only the crystal structures of sodium fenoprofenate dihydrate (Stephenson & Diseroad, 2000) and calcium fenoprofenate monohydrate (Zhu *et al.*, 2001) have been reported. On the other hand, the complexes of copper(II) with other nonsteroidal anti-inflammatory drugs (NSAIDs) have been widely studied because they have enhanced anti-inflammatory activity and reduced gastrointestinal toxicity compared with their uncomplexed parent drugs (Weder *et al.*, 2002).

Aiming to contribute to the knowledge of new fenoprofen coordination compounds of biological interest, we report in this paper the crystal structure of the complex Cu_2_(Fen)_4_(DMF)_2_(Fen: fenoprofenate anion, C_15_H_13_O_3_^-^; DMF: dimethylformamide).

Each Cu(II) ion in the dinuclear complex (Fig. 1)has four oxygen atoms from different carboxylate groups in equatorial positions, with a Cu···O range of 1.95–1.96 Å. The square pyramidal geometry is completed with an oxygen atom from the DMF molecule with a Cu—O distance of 2.1634 (39) Å. The four carboxylate bridges linking copper ions form the classical *paddle-wheel* type cage with a Cu···Cu distance of 2.6309 (20) Å. The intermetallic separation is in the range of reported distances for other dicopper(II) tetracarboxilates (Weder *et al.*, 2002). The two enantiomeric forms of the fenoprofenate anion are present in the complex, in an optically inactive centrosymmetric arrangement, where atoms C12 and C32 (shown in Fig. 1) correspond to the *R*-enantiomer.

There are no conventional hydrogen bonds in the structure; while weak π···π and C—H···πi interactions were identified. In the case of the π···π interactions, one of them corresponds to symmetry related phenyl rings (C14—C19, centroid *Cg*1)with a *Cg*1-*Cg*1^i^ distance of 3.620 (4) Å [symmetry code (i) = 1 - *x*, 3 - *y*, -*z*] , a dihedral angle α=0°, an a slippage angle β = 18.22° .

The other intermolecular phenyl interaction corresponds to C20—C25 (centroid *Cg*2) and C40—C45 (centroid *Cg*3)rings, the *Cg*2-*Cg3*^ii^ distance being 4.132 (6) Å [symmetry code (ii) = 1 - *x*, 1/2 + *y*,1/2 - *z*], with a dihedral angle α= 22.1 (4)°, and β=23.63°.

Additionally C14—C19 and C40—C45 rings are involved in intermolecular C—H···πi interactions. The C22—H22···*Cg*1^iii ^angle is 160° [symmetry code (iii) = 2 - *x*, 3 - *y*, -*z*] and the H22···*Cg*1^iii^distance is 2.78 Å. The C24—H24···*Cg3*^iv^ angle is 147° [symmetry code (iv) = 1 + *x*,*y*, *z*] and the H24···*Cg3*^iv^ distance is 2.73 Å.

Although the steric demand from the benzyl groups avoids significant contact between neighbour binuclear units, the intermolecular interactions form a weak two-dimensional network parallel to the (100) plane (Fig. 2).

### Experimental

A 2.0 mLDMF solution containing 0.0810 g (0.150 mmol) of racemic fenoprofen calcium salthydrate (Ca(Fen)_2_.H_2_O) was added to a 3.0 ml e thanolicsolution of 0.0170 g (0.100 mmol) CuCl_2_.2H_2_O.The resulting green solution was stirred at room temperature for about one hourand reposed all over the night. Then an excess of water was added leading tothe immediate precipitation of the complex, which gradually recrystallized atroom temperature after three weeks. The bright green crystals obtained werefiltered, washed with water and air dried (Yield: 43%). Analysis calculated for C_66_H_66_Cu_2_N_2_O_14_:*C* 64.02, H 5.37, N 2.26%. Found: C 63.5, H 4.9, N, 1.7%. FTIR(cm^-1^): 1668 (DMF), 1614 (*asym*. stretch.COO), 1484 (*sym*. stretch. COO). UV-visible (DMF, λmax/nm): 705.

### Refinement

The H atoms were positionedgeometrically and treated as riding with C—H = 0.93–0.98 Å. H atoms bonded totertiary C atoms were refined with *U*_iso_(H)=1.2*U*_eq_(C),while for the rest *U*_iso_(H)=1.5*U*_eq_(C). Additionally, the idealized H atoms from the DMF's methyl groups were allowed to ride on the immediate Catoms. The anisotropic displacements ellipsoids of atoms O13 and O33 displayeda marked elongation in the normal direction to both O—C bond directions,indicating a possible conformation disorder. It can be related with thetendency of higher values of *U*_eq_ for the outermost phenyl carbonatoms.

The position of the highest residual electron-density peak is located at 1.67 Åfrom N52.

### Crystal data

**Table:**

[Cu_2_(C_15_H_13_O_3_)_4_(C_3_H_7_NO)_2_]	*F*_000_ = 1292
*M**_r_* = 1238.29	*D*_x_ = 1.354 Mg m^−^^3^
Monoclinic, *P*2_1_/*c*	Mo *K*α radiation λ = 0.71073 Å
Hall symbol: -P 2ybc	Cell parameters from 50 reflections
*a* = 11.142 (8) Å	θ = 5.1–13.6º
*b* = 11.580 (8) Å	µ = 0.77 mm^−^^1^
*c* = 23.891 (6) Å	*T* = 293 (2) K
β = 99.85 (6)º	Plate, green
*V* = 3037 (3) Å^3^	0.20 × 0.20 × 0.10 mm
*Z* = 2	

### Data collection

**Table:**

Rigaku AFC-7S diffractometer	*R*_int_ = 0.091
Radiation source: fine-focus sealed tube	θ_max_ = 27.5º
Monochromator: graphite	θ_min_ = 2.3º
*T* = 293(2) K	*h* = −1→14
θ/2θ scans	*k* = −1→15
Absorption correction: ψ scan(North *et al.*, 1968)	*l* = −31→30
*T*_min_ = 0.862, *T*_max_ = 0.927	3 standard reflections
8818 measured reflections	every 150 reflections
6969 independent reflections	intensity decay: none
4101 reflections with *I* > 2σ(*I*)	

### Refinement

**Table:**

Refinement on *F*^2^	Secondary atom site location: difference Fourier map
Least-squares matrix: full	Hydrogen site location: inferred from neighbouring sites
*R*[*F*^2^ > 2σ(*F*^2^)] = 0.064	H-atom parameters constrained
*wR*(*F*^2^) = 0.200	*w* = 1/[σ^2^(*F*_o_^2^) + (0.0755*P*)^2^ + 0.3252*P*] where *P* = (*F*_o_^2^ + 2*F*_c_^2^)/3
*S* = 1.02	(Δ/σ)_max_ < 0.001
6969 reflections	Δρ_max_ = 0.51 e Å^−^^3^
381 parameters	Δρ_min_ = −0.54 e Å^−^^3^
Primary atom site location: structure-invariant direct methods	Extinction correction: none

### Special details

**Table:**

Geometry. All e.s.d.'s (except the e.s.d. in the dihedral angle between two l.s. planes) are estimated using the full covariance matrix. The cell e.s.d.'s are taken into account individually in the estimation of e.s.d.'s in distances, angles and torsion angles; correlations between e.s.d.'s in cell parameters are only used when they are defined by crystal symmetry. An approximate (isotropic) treatment of cell e.s.d.'s is used for estimating e.s.d.'s involving l.s. planes.

### Fractional atomic coordinates and isotropic or equivalent isotropic displacement parameters (Å^2^)

**Table:**

	*x*	*y*	*z*	*U*_iso_*/*U*_eq_	
C11	0.5569 (6)	1.1752 (5)	−0.0520 (2)	0.0417 (15)	
C12	0.5867 (6)	1.2834 (6)	−0.0844 (3)	0.0546 (17)	
H12	0.5103	1.3066	−0.1084	0.066*	
C13	0.6736 (8)	1.2579 (7)	−0.1238 (3)	0.093 (3)	
H13A	0.6440	1.1936	−0.1476	0.139*	
H13B	0.7520	1.2393	−0.1022	0.139*	
H13C	0.6808	1.3244	−0.1470	0.139*	
C14	0.6237 (5)	1.3829 (5)	−0.0446 (2)	0.0391 (13)	
C15	0.5925 (5)	1.4966 (5)	−0.0623 (3)	0.0511 (17)	
H15	0.5477	1.5090	−0.0983	0.061*	
C16	0.6263 (6)	1.5889 (6)	−0.0277 (3)	0.0599 (19)	
H16	0.6059	1.6631	−0.0409	0.072*	
C17	0.6902 (6)	1.5738 (6)	0.0264 (4)	0.061 (2)	
H17	0.7133	1.6368	0.0499	0.073*	
C18	0.7191 (5)	1.4632 (6)	0.0449 (3)	0.0501 (15)	
C19	0.6876 (5)	1.3686 (5)	0.0097 (3)	0.0413 (14)	
H19	0.7098	1.2948	0.0230	0.050*	
C20	0.9021 (5)	1.4495 (5)	0.1130 (3)	0.0461 (15)	
C21	0.9731 (6)	1.4870 (7)	0.0754 (3)	0.066 (2)	
H21	0.9375	1.5124	0.0395	0.079*	
C22	1.0966 (6)	1.4868 (9)	0.0911 (3)	0.095 (3)	
H22	1.1453	1.5098	0.0652	0.114*	
C23	1.1509 (7)	1.4529 (8)	0.1453 (4)	0.088 (3)	
H23	1.2353	1.4540	0.1557	0.106*	
C24	1.0802 (7)	1.4184 (7)	0.1828 (3)	0.071 (2)	
H24	1.1159	1.3953	0.2191	0.085*	
C25	0.9548 (6)	1.4174 (6)	0.1672 (3)	0.0572 (17)	
H25	0.9061	1.3951	0.1933	0.069*	
C31	0.5588 (6)	1.0976 (6)	0.0955 (2)	0.0477 (15)	
C32	0.5866 (6)	1.1560 (7)	0.1538 (3)	0.062 (2)	
H32	0.5509	1.2335	0.1490	0.075*	
C33	0.7150 (8)	1.1719 (9)	0.1752 (3)	0.109 (4)	
H33A	0.7515	1.2141	0.1479	0.163*	
H33B	0.7537	1.0979	0.1817	0.163*	
H33C	0.7250	1.2142	0.2102	0.163*	
C34	0.5154 (6)	1.0908 (7)	0.1932 (3)	0.0566 (17)	
C35	0.3948 (6)	1.1190 (7)	0.1930 (3)	0.0602 (19)	
H35	0.3581	1.1753	0.1679	0.072*	
C36	0.3279 (6)	1.0659 (8)	0.2289 (3)	0.071 (2)	
C37	0.3784 (8)	0.9814 (9)	0.2655 (3)	0.092 (3)	
H37	0.3338	0.9470	0.2906	0.111*	
C38	0.4965 (8)	0.9486 (9)	0.2645 (4)	0.097 (3)	
H38	0.5312	0.8889	0.2878	0.116*	
C39	0.5636 (7)	1.0035 (7)	0.2291 (3)	0.074 (2)	
H39	0.6439	0.9809	0.2295	0.088*	
C40	0.1689 (6)	1.1642 (7)	0.2655 (3)	0.0594 (18)	
C41	0.2497 (7)	1.2272 (7)	0.3036 (3)	0.074 (2)	
H41	0.3331	1.2224	0.3037	0.089*	
C42	0.2046 (10)	1.2973 (8)	0.3415 (3)	0.095 (3)	
H42	0.2578	1.3406	0.3676	0.114*	
C43	0.0822 (11)	1.3038 (10)	0.3410 (4)	0.108 (4)	
H43	0.0522	1.3519	0.3665	0.129*	
C44	0.0026 (8)	1.2392 (9)	0.3027 (4)	0.088 (3)	
H44	−0.0806	1.2425	0.3031	0.106*	
C45	0.0460 (7)	1.1709 (7)	0.2645 (3)	0.068 (2)	
H45	−0.0074	1.1290	0.2379	0.082*	
C51	0.8413 (6)	0.9038 (6)	−0.0378 (3)	0.0563 (17)	
H51	0.7959	0.9392	−0.0694	0.068*	
C53	1.0207 (7)	0.8041 (8)	0.0057 (3)	0.094 (3)	
H53A	1.0998	0.8390	0.0148	0.141*	
H53B	1.0293	0.7246	−0.0044	0.141*	
H53C	0.9804	0.8086	0.0380	0.141*	
C54	1.0004 (8)	0.8783 (9)	−0.0945 (3)	0.098 (3)	
H54A	0.9425	0.9156	−0.1231	0.147*	
H54B	1.0199	0.8034	−0.1077	0.147*	
H54C	1.0732	0.9241	−0.0866	0.147*	
Cu1	0.61251 (6)	0.96206 (6)	0.00433 (3)	0.0391 (2)	
O11	0.6419 (4)	1.1044 (4)	−0.03595 (19)	0.0546 (12)	
O12	0.4481 (4)	1.1667 (3)	−0.04371 (17)	0.0468 (10)	
O13	0.7767 (4)	1.4439 (4)	0.10133 (18)	0.0607 (13)	
O31	0.6426 (4)	1.0452 (4)	0.07660 (17)	0.0529 (10)	
O32	0.4517 (4)	1.1100 (4)	0.06887 (18)	0.0536 (11)	
O33	0.2043 (4)	1.0911 (6)	0.2244 (2)	0.095 (2)	
O51	0.7945 (4)	0.8967 (4)	0.00567 (19)	0.0579 (12)	
N52	0.9482 (5)	0.8656 (5)	−0.0425 (3)	0.0612 (16)	

### Atomic displacement parameters (Å^2^)

**Table:**

	*U*^11^	*U*^22^	*U*^33^	*U*^12^	*U*^13^	*U*^23^
C11	0.064 (4)	0.032 (3)	0.029 (3)	−0.018 (3)	0.009 (3)	−0.001 (2)
C12	0.063 (4)	0.052 (4)	0.049 (4)	−0.013 (3)	0.011 (3)	0.008 (3)
C13	0.138 (8)	0.065 (5)	0.084 (6)	−0.028 (6)	0.046 (6)	0.014 (5)
C14	0.027 (3)	0.038 (3)	0.053 (4)	−0.001 (3)	0.009 (3)	0.007 (3)
C15	0.026 (3)	0.048 (4)	0.079 (5)	0.000 (3)	0.008 (3)	0.017 (3)
C16	0.050 (4)	0.038 (4)	0.092 (6)	0.001 (3)	0.013 (4)	0.009 (4)
C17	0.055 (4)	0.040 (4)	0.092 (6)	0.001 (3)	0.025 (4)	−0.009 (4)
C18	0.029 (3)	0.057 (4)	0.068 (4)	−0.007 (4)	0.019 (3)	−0.004 (4)
C19	0.025 (3)	0.038 (3)	0.062 (4)	0.003 (3)	0.011 (3)	0.009 (3)
C20	0.031 (3)	0.046 (4)	0.061 (4)	−0.009 (3)	0.007 (3)	−0.004 (3)
C21	0.041 (3)	0.092 (6)	0.061 (4)	−0.011 (4)	0.001 (3)	0.014 (4)
C22	0.043 (4)	0.170 (10)	0.074 (5)	−0.021 (5)	0.018 (4)	0.034 (6)
C23	0.045 (4)	0.123 (8)	0.088 (6)	−0.001 (5)	−0.010 (4)	0.024 (6)
C24	0.066 (5)	0.070 (5)	0.070 (5)	−0.002 (4)	−0.005 (4)	0.022 (4)
C25	0.059 (4)	0.057 (4)	0.057 (4)	−0.011 (3)	0.011 (3)	0.001 (3)
C31	0.058 (4)	0.053 (4)	0.033 (3)	−0.020 (4)	0.012 (3)	−0.005 (3)
C32	0.058 (4)	0.084 (5)	0.048 (4)	−0.020 (4)	0.017 (3)	−0.020 (4)
C33	0.113 (8)	0.145 (9)	0.067 (6)	−0.066 (7)	0.013 (5)	−0.019 (6)
C34	0.061 (4)	0.069 (4)	0.039 (4)	−0.009 (4)	0.005 (3)	−0.013 (3)
C35	0.065 (4)	0.078 (5)	0.036 (4)	0.000 (4)	0.002 (3)	−0.008 (3)
C36	0.052 (4)	0.111 (7)	0.050 (4)	0.011 (4)	0.009 (4)	−0.013 (4)
C37	0.074 (5)	0.141 (9)	0.065 (5)	0.002 (6)	0.023 (4)	0.036 (6)
C38	0.076 (6)	0.124 (9)	0.090 (6)	0.014 (6)	0.016 (5)	0.032 (6)
C39	0.054 (4)	0.095 (6)	0.076 (5)	0.007 (4)	0.021 (4)	0.002 (5)
C40	0.050 (4)	0.074 (5)	0.054 (4)	−0.002 (4)	0.006 (3)	0.009 (4)
C41	0.064 (5)	0.091 (6)	0.062 (5)	0.000 (5)	−0.004 (4)	0.007 (5)
C42	0.117 (8)	0.097 (7)	0.063 (6)	0.011 (6)	−0.007 (5)	−0.003 (5)
C43	0.136 (9)	0.123 (9)	0.072 (6)	0.044 (8)	0.040 (7)	−0.008 (6)
C44	0.082 (6)	0.112 (8)	0.075 (6)	0.019 (6)	0.023 (5)	0.012 (6)
C45	0.059 (4)	0.081 (5)	0.067 (5)	0.000 (4)	0.015 (4)	0.005 (4)
C51	0.043 (4)	0.063 (4)	0.060 (4)	0.002 (4)	0.002 (3)	−0.002 (4)
C53	0.063 (5)	0.108 (7)	0.111 (7)	0.041 (5)	0.015 (5)	0.015 (6)
C54	0.091 (6)	0.122 (8)	0.092 (6)	0.009 (6)	0.047 (5)	−0.012 (6)
Cu1	0.0343 (3)	0.0397 (4)	0.0429 (4)	0.0012 (4)	0.0060 (3)	−0.0011 (4)
O11	0.037 (2)	0.047 (3)	0.081 (3)	0.001 (2)	0.013 (2)	0.012 (2)
O12	0.043 (2)	0.037 (2)	0.061 (3)	−0.0041 (19)	0.010 (2)	0.013 (2)
O13	0.039 (2)	0.085 (4)	0.058 (3)	−0.014 (2)	0.010 (2)	−0.009 (3)
O31	0.049 (2)	0.058 (3)	0.051 (2)	−0.008 (2)	0.004 (2)	−0.013 (2)
O32	0.046 (2)	0.062 (3)	0.052 (3)	−0.004 (2)	0.003 (2)	−0.011 (2)
O33	0.056 (3)	0.147 (6)	0.082 (4)	0.005 (3)	0.009 (3)	−0.042 (4)
O51	0.042 (2)	0.071 (3)	0.062 (3)	0.018 (2)	0.014 (2)	0.004 (3)
N52	0.038 (3)	0.069 (4)	0.080 (4)	0.008 (3)	0.022 (3)	−0.017 (3)

### Geometric parameters (Å, °)

**Table:**

C11—O11	1.261 (7)	C34—C35	1.382 (9)
C11—O12	1.265 (7)	C35—C36	1.375 (9)
C11—C12	1.538 (8)	C35—H35	0.9300
C12—C13	1.491 (9)	C36—C37	1.368 (11)
C12—C14	1.505 (8)	C36—O33	1.393 (8)
C12—H12	0.9800	C37—C38	1.374 (11)
C13—H13A	0.9600	C37—H37	0.9300
C13—H13B	0.9600	C38—C39	1.378 (10)
C13—H13C	0.9600	C38—H38	0.9300
C14—C19	1.381 (8)	C39—H39	0.9300
C14—C15	1.407 (8)	C40—C45	1.368 (9)
C15—C16	1.364 (9)	C40—C41	1.375 (10)
C15—H15	0.9300	C40—O33	1.403 (8)
C16—C17	1.377 (10)	C41—C42	1.374 (11)
C16—H16	0.9300	C41—H41	0.9300
C17—C18	1.375 (9)	C42—C43	1.364 (12)
C17—H17	0.9300	C42—H42	0.9300
C18—C19	1.388 (8)	C43—C44	1.379 (12)
C18—O13	1.408 (7)	C43—H43	0.9300
C19—H19	0.9300	C44—C45	1.357 (10)
C20—C21	1.364 (9)	C44—H44	0.9300
C20—O13	1.379 (7)	C45—H45	0.9300
C20—C25	1.379 (8)	C51—O51	1.241 (7)
C21—C22	1.364 (9)	C51—N52	1.293 (8)
C21—H21	0.9300	C51—H51	0.9300
C22—C23	1.388 (10)	C53—N52	1.472 (9)
C22—H22	0.9300	C53—H53A	0.9600
C23—C24	1.351 (10)	C53—H53B	0.9600
C23—H23	0.9300	C53—H53C	0.9600
C24—C25	1.383 (10)	C54—N52	1.465 (8)
C24—H24	0.9300	C54—H54A	0.9600
C25—H25	0.9300	C54—H54B	0.9600
C31—O31	1.260 (7)	C54—H54C	0.9600
C31—O32	1.260 (7)	Cu1—O12^i^	1.945 (4)
C31—C32	1.533 (8)	Cu1—O31	1.955 (4)
C32—C33	1.446 (10)	Cu1—O32^i^	1.959 (4)
C32—C34	1.530 (9)	Cu1—O11	1.964 (4)
C32—H32	0.9800	Cu1—O51	2.160 (4)
C33—H33A	0.9600	Cu1—Cu1^i^	2.631 (2)
C33—H33B	0.9600	O12—Cu1^i^	1.945 (4)
C33—H33C	0.9600	O32—Cu1^i^	1.959 (4)
C34—C39	1.373 (10)		
			
O11—C11—O12	126.3 (5)	C34—C35—H35	119.3
O11—C11—C12	117.8 (5)	C37—C36—C35	120.8 (7)
O12—C11—C12	115.9 (6)	C37—C36—O33	119.5 (7)
C13—C12—C14	114.4 (6)	C35—C36—O33	119.5 (7)
C13—C12—C11	112.1 (6)	C36—C37—C38	118.5 (8)
C14—C12—C11	111.4 (5)	C36—C37—H37	120.8
C13—C12—H12	106.1	C38—C37—H37	120.8
C14—C12—H12	106.1	C37—C38—C39	120.4 (8)
C11—C12—H12	106.1	C37—C38—H38	119.8
C12—C13—H13A	109.5	C39—C38—H38	119.8
C12—C13—H13B	109.5	C34—C39—C38	121.8 (7)
H13A—C13—H13B	109.5	C34—C39—H39	119.1
C12—C13—H13C	109.5	C38—C39—H39	119.1
H13A—C13—H13C	109.5	C45—C40—C41	121.4 (8)
H13B—C13—H13C	109.5	C45—C40—O33	115.0 (7)
C19—C14—C15	117.0 (6)	C41—C40—O33	123.6 (7)
C19—C14—C12	122.9 (5)	C40—C41—C42	118.6 (8)
C15—C14—C12	120.0 (5)	C40—C41—H41	120.7
C16—C15—C14	121.6 (6)	C42—C41—H41	120.7
C16—C15—H15	119.2	C43—C42—C41	120.2 (9)
C14—C15—H15	119.2	C43—C42—H42	119.9
C15—C16—C17	120.9 (7)	C41—C42—H42	119.9
C15—C16—H16	119.5	C42—C43—C44	120.3 (9)
C17—C16—H16	119.5	C42—C43—H43	119.8
C18—C17—C16	118.4 (7)	C44—C43—H43	119.8
C18—C17—H17	120.8	C45—C44—C43	119.9 (9)
C16—C17—H17	120.8	C45—C44—H44	120.0
C17—C18—C19	121.3 (6)	C43—C44—H44	120.0
C17—C18—O13	119.9 (7)	C44—C45—C40	119.5 (8)
C19—C18—O13	118.7 (6)	C44—C45—H45	120.2
C14—C19—C18	120.7 (6)	C40—C45—H45	120.2
C14—C19—H19	119.6	O51—C51—N52	125.2 (7)
C18—C19—H19	119.6	O51—C51—H51	117.4
C21—C20—O13	124.3 (6)	N52—C51—H51	117.4
C21—C20—C25	120.1 (6)	N52—C53—H53A	109.5
O13—C20—C25	115.5 (5)	N52—C53—H53B	109.5
C22—C21—C20	119.3 (7)	H53A—C53—H53B	109.5
C22—C21—H21	120.3	N52—C53—H53C	109.5
C20—C21—H21	120.3	H53A—C53—H53C	109.5
C21—C22—C23	121.1 (7)	H53B—C53—H53C	109.5
C21—C22—H22	119.5	N52—C54—H54A	109.5
C23—C22—H22	119.5	N52—C54—H54B	109.5
C24—C23—C22	119.4 (7)	H54A—C54—H54B	109.5
C24—C23—H23	120.3	N52—C54—H54C	109.5
C22—C23—H23	120.3	H54A—C54—H54C	109.5
C23—C24—C25	120.0 (7)	H54B—C54—H54C	109.5
C23—C24—H24	120.0	O12^i^—Cu1—O31	88.38 (18)
C25—C24—H24	120.0	O12^i^—Cu1—O32^i^	90.05 (18)
C20—C25—C24	119.9 (6)	O31—Cu1—O32^i^	168.30 (17)
C20—C25—H25	120.0	O12^i^—Cu1—O11	168.87 (17)
C24—C25—H25	120.0	O31—Cu1—O11	90.08 (19)
O31—C31—O32	124.7 (5)	O32^i^—Cu1—O11	89.23 (19)
O31—C31—C32	119.4 (6)	O12^i^—Cu1—O51	97.32 (18)
O32—C31—C32	115.8 (6)	O31—Cu1—O51	98.09 (18)
C33—C32—C34	115.6 (6)	O32^i^—Cu1—O51	93.61 (18)
C33—C32—C31	114.3 (6)	O11—Cu1—O51	93.81 (17)
C34—C32—C31	107.2 (5)	O12^i^—Cu1—Cu1^i^	83.62 (13)
C33—C32—H32	106.4	O31—Cu1—Cu1^i^	85.52 (14)
C34—C32—H32	106.4	O32^i^—Cu1—Cu1^i^	82.78 (14)
C31—C32—H32	106.4	O11—Cu1—Cu1^i^	85.28 (13)
C32—C33—H33A	109.5	O51—Cu1—Cu1^i^	176.28 (13)
C32—C33—H33B	109.5	C11—O11—Cu1	121.0 (4)
H33A—C33—H33B	109.5	C11—O12—Cu1^i^	123.8 (4)
C32—C33—H33C	109.5	C20—O13—C18	117.8 (5)
H33A—C33—H33C	109.5	C31—O31—Cu1	121.9 (4)
H33B—C33—H33C	109.5	C31—O32—Cu1^i^	124.8 (4)
C39—C34—C35	116.9 (7)	C36—O33—C40	117.7 (6)
C39—C34—C32	124.0 (7)	C51—O51—Cu1	119.5 (4)
C35—C34—C32	119.1 (7)	C51—N52—C54	123.0 (7)
C36—C35—C34	121.5 (7)	C51—N52—C53	119.1 (6)
C36—C35—H35	119.3	C54—N52—C53	117.8 (6)
			
O11—C11—C12—C13	37.9 (8)	C32—C34—C39—C38	178.9 (7)
O12—C11—C12—C13	−142.6 (6)	C37—C38—C39—C34	−1.1 (14)
O11—C11—C12—C14	−91.8 (7)	C45—C40—C41—C42	0.5 (12)
O12—C11—C12—C14	87.8 (6)	O33—C40—C41—C42	179.1 (7)
C13—C12—C14—C19	−94.5 (7)	C40—C41—C42—C43	−0.1 (13)
C11—C12—C14—C19	33.9 (8)	C41—C42—C43—C44	0.5 (15)
C13—C12—C14—C15	85.9 (8)	C42—C43—C44—C45	−1.5 (15)
C11—C12—C14—C15	−145.7 (6)	C43—C44—C45—C40	1.9 (13)
C19—C14—C15—C16	1.5 (9)	C41—C40—C45—C44	−1.5 (12)
C12—C14—C15—C16	−178.8 (6)	O33—C40—C45—C44	179.9 (7)
C14—C15—C16—C17	−1.5 (10)	O12—C11—O11—Cu1	1.8 (8)
C15—C16—C17—C18	−0.1 (10)	C12—C11—O11—Cu1	−178.7 (4)
C16—C17—C18—C19	1.5 (9)	O12^i^—Cu1—O11—C11	−4.9 (13)
C16—C17—C18—O13	−175.5 (5)	O31—Cu1—O11—C11	−86.9 (5)
C15—C14—C19—C18	−0.1 (8)	O32^i^—Cu1—O11—C11	81.4 (5)
C12—C14—C19—C18	−179.7 (5)	O51—Cu1—O11—C11	175.0 (5)
C17—C18—C19—C14	−1.5 (8)	O11—C11—O12—Cu1^i^	−0.9 (8)
O13—C18—C19—C14	175.6 (4)	C12—C11—O12—Cu1^i^	179.6 (4)
O13—C20—C21—C22	−178.8 (8)	C21—C20—O13—C18	9.0 (10)
C25—C20—C21—C22	3.3 (12)	C25—C20—O13—C18	−173.0 (6)
C20—C21—C22—C23	−2.2 (14)	C17—C18—O13—C20	−87.2 (7)
C21—C22—C23—C24	0.7 (15)	C19—C18—O13—C20	95.8 (6)
C22—C23—C24—C25	−0.2 (14)	O32—C31—O31—Cu1	−5.8 (9)
C21—C20—C25—C24	−2.8 (11)	C32—C31—O31—Cu1	176.9 (4)
O13—C20—C25—C24	179.0 (7)	O12^i^—Cu1—O31—C31	−81.3 (5)
C23—C24—C25—C20	1.3 (12)	O32^i^—Cu1—O31—C31	1.1 (12)
O31—C31—C32—C33	16.0 (10)	O11—Cu1—O31—C31	87.7 (5)
O32—C31—C32—C33	−161.6 (7)	O51—Cu1—O31—C31	−178.4 (5)
O31—C31—C32—C34	−113.4 (7)	O31—C31—O32—Cu1^i^	6.3 (9)
O32—C31—C32—C34	69.0 (8)	C32—C31—O32—Cu1^i^	−176.2 (4)
C33—C32—C34—C39	−33.3 (11)	C37—C36—O33—C40	81.8 (10)
C31—C32—C34—C39	95.4 (8)	C35—C36—O33—C40	−103.9 (8)
C33—C32—C34—C35	147.4 (7)	C45—C40—O33—C36	−169.9 (7)
C31—C32—C34—C35	−84.0 (8)	C41—C40—O33—C36	11.5 (11)
C39—C34—C35—C36	2.9 (10)	N52—C51—O51—Cu1	−178.4 (5)
C32—C34—C35—C36	−177.7 (6)	O12^i^—Cu1—O51—C51	136.1 (5)
C34—C35—C36—C37	−1.2 (12)	O31—Cu1—O51—C51	−134.5 (5)
C34—C35—C36—O33	−175.4 (7)	O32^i^—Cu1—O51—C51	45.6 (5)
C35—C36—C37—C38	−1.8 (14)	O11—Cu1—O51—C51	−43.9 (5)
O33—C36—C37—C38	172.4 (8)	O51—C51—N52—C54	−178.6 (7)
C36—C37—C38—C39	2.9 (15)	O51—C51—N52—C53	2.8 (11)
C35—C34—C39—C38	−1.8 (12)		

Symmetry codes: (i) −*x*+1, −*y*+2, −*z*.
